# Impact of Delaying the Addition of Anti-EGFR in First Line of RAS Wild-Type Metastatic Colorectal Cancer: A Propensity-Weighted Pooled Data Analysis

**DOI:** 10.3390/cancers14061410

**Published:** 2022-03-10

**Authors:** Lola-Jade Palmieri, Tomas Buchler, Antoine Meyer, Veronika Veskrnova, Ondrej Fiala, Petr Brabec, Jana Baranova, Romain Coriat

**Affiliations:** 1 Gastroenterology and Digestive Oncology Department, Cochin Hospital, AP-HP, 27 rue du Faubourg Saint Jacques, 75014 Paris, France; l.palmieri@bordeaux.unicancer.fr; 2 Faculté de Médecine Paris Centre, Université de Paris, 75006 Paris, France; 3 Department of Oncology, First Faculty of Medicine, Charles University and Thomayer University Hospital, Videnska 800, 14059 Prague, Czech Republic; tomas.buchler@ftn.cz (T.B.); veskrnova.ver@gmail.com (V.V.); 4 Hôpital Bicêtre, AP-HP, 78 rue du General Leclerc, 94043 Le Kremlin Bicêtre, France; antoine.meyer@aphp.fr; 5 Faculté de Médecine, Université Paris Saclay, 94043 Le Kremlin Bicêtre, France; 6 Department of Oncology and Radiotherapeutics, Faculty of Medicine and University Hospital in Pilsen, Charles University, alej Svobody 80, 32300 Pilsen, Czech Republic; fiala.o@centrum.cz; 7 Biomedical Center, Faculty of Medicine in Pilsen, Charles University, 32300 Pilsen, Czech Republic; 8 Institute of Biostatistics and Analyses, Faculty of Medicine, Masaryk University, Netroufalky 5, 62500 Brno, Czech Republic; brabec@biostatistika.cz (P.B.); baranova@biostatistika.cz (J.B.)

**Keywords:** metastatic colorectal cancer, *RAS* status, anti-EGFR, bevacizumab

## Abstract

**Simple Summary:**

The first-line therapy of patients with RAS wild-type (WT) non-resectable metastatic colorectal cancer (mCRC) is usually 5-fluorouracil-based chemotherapy with either bevacizumab or an anti-epidermal growth factor receptor (EGFR). The introduction of anti-epidermal growth factor antibodies is commonly delayed because of late RAS testing results. Our objective was to evaluate the impact on the overall survival of delayed anti-EGFR introduction strategy. This study compared 305 patients with delayed anti-EGFR introductions, 401 with immediate anti-EGFRs, and 129 with immediate anti-VEGFs. The study suggests that delayed introduction has no deleterious impact on survival compared to the immediate introduction of an anti-EGFR or of an anti-VEGF.

**Abstract:**

The first-line therapy of patients with RAS wild-type (WT) non-resectable metastatic colorectal cancer (mCRC) is usually 5-fluorouracil-based chemotherapy with either bevacizumab or an anti-epidermal growth factor receptor (EGFR). The addition of anti-EGFR antibodies is commonly delayed in clinical practice because of late RAS testing results. Our objective was to evaluate the impact on overall survival (OS) of a delayed anti-EGFR introduction strategy. This study pooled the data of two large retrospective studies. Patients with RAS WT non-resectable mCRC, treated in first line by a doublet chemotherapy with an anti-EGFR introduced with a delay of 2 to 4 cycles, were compared to an anti-EGFR and to an anti-VEGF that was introduced immediately. Patients numbering 305 in the delayed anti-EGFR group, 401 in the immediate anti-EGFR group, and 129 in the immediate anti-VEGF group were analyzed. After propensity scoring, there was no difference between the characteristics of the three groups. Median OS was 28.6 months (95% CI: 23.5–34.1) in the immediate anti-EGFR group, 35.1 (95% CI: 29.9–43.5) in the delayed anti-EGFR group, and 32.4 (95% CI: 25.4–44.8) in the immediate anti-VEGF group. There was no significant difference concerning median OS (*p* = 0.24) or progression-free survival (*p* = 0.56). This study suggests that delaying the introduction of an anti-EGFR has no deleterious impact on survival compared to the immediate introduction of an anti-VEGF or of an anti-EGFR.

## 1. Introduction

Colorectal cancer was the third most frequent cancer and the second cause of cancer-related mortality worldwide in 2020, with an estimated mortality rate of up to 577,000 deaths per year [1]. Based on FIRE-3 and CALGB/SWOG 80405 phase III trials, guidelines propose, as the main treatment option in first-line *RAS*/*BRAF* wild-type (WT) non-resectable metastatic colorectal cancer (mCRC), 5FU-based doublet chemotherapy with oxaliplatin (FOLFOX) or irinotecan (FOLFIRI), in combination with either an anti-epidermal growth factor receptor monoclonal antibody (anti-EGFR), cetuximab or panitumumab, or an anti-vascular endothelial growth factor monoclonal antibody (anti-VEGF), bevacizumab [2,3,4]. 

More than half of mCRC have a *RAS* mutation (*KRAS* exon 2,3,4 or *NRAS* exon 2,3,4) [5]. The high predictive value of *KRAS* mutations in response to cetuximab was proved in 2008 [6]. This was later confirmed and completed with extended mutations in *KRAS* and *NRAS* exons 2, 3, and 4 [7,8,9]. These results led to the recommendation of obtaining *RAS* status before the introduction of an anti-EGFR in first-line chemotherapy for *RAS* WT mCRC. The median time for the results of *KRAS* and *NRAS* status was evaluated in 2014 to be 20 days [10]. Thus, it is common that the addition of anti-EGFR antibodies might be delayed. In routine practice, oncologists often initiate doublet chemotherapy without any monoclonal antibody and subsequently introduce anti-EGFR when the WT *RAS* status is available. Another option is not to wait and use doublet chemotherapy with bevacizumab up front.

Two retrospective studies including patients with *KRAS/NRAS* WT mCRC recently assessed this issue [11,12]. One compared the anti-EGFR added to chemotherapy simultaneously with the administration of the first cycle, versus the anti-EGFR added to chemotherapy at cycle 2, and versus the anti-EGFR added to the chemotherapy at cycle 3 or 4 and found no difference concerning overall survival (OS) nor progression-free survival (PFS) in the three groups [11]. The other study compared the anti-VEGF started at first cycle versus the anti-EGFR started at cycle 2 or 3, in combination with doublet chemotherapy, and found no difference between the groups concerning OS [12]. Of note, *BRAF* status was unknown for more than half of the first cohort.

The present study pooled the data of these two studies, with a propensity score weighting, to compare the three strategies and evaluate the impact of delaying anti-EGFR introduction on OS compared with an immediate anti-EGFR introduction and compared with an immediate anti-VEGF introduction. 

## 2. Methods

This study pooled the data of two retrospective studies. The first one was a Czech retrospective cohort on a national data registry of 573 patients with *RAS* WT mCRC, treated in first line with anti-EGFR and doublet chemotherapy between 2010 and 2017 [11]. Three groups were compared: anti-EGFR added to chemotherapy at first cycle (N = 401), anti-EGFR added to chemotherapy at cycle 2 (N = 71), and anti-EGFR added to the chemotherapy at cycle 3 or 4 (N = 101). Of note, this study did not include patients with early disease progression during first-line chemotherapy and those who did not tolerate chemotherapy before the addition of an anti-EGFR. The second study was a French multicenter retrospective analysis on 262 patients with *RAS* WT mCRC, treated in first line with an anti-EGFR or anti-VEGF and doublet chemotherapy between 2013 and 2016 [12]. The immediate introduction of an anti-VEGF started at first cycle (N = 129) was compared to the delayed introduction of an anti-EGFR started at cycle 2 or 3 (N = 133). The present study pooled this data to compare three groups: immediate anti-EGFR introduction (N = 401), delayed anti-EGFR introduction between cycle 2 and 4 (N = 305), and immediate anti-VEGF introduction (N = 129).

The primary outcome was OS, which is defined as the time from the first cycle of chemotherapy to death from any cause. Patients lost to follow-up or were alive at the time of the last assessment were censored. Secondary outcomes included PFS and objective response rate (ORR). PFS was defined as the time from the first cycle of chemotherapy to disease progression or death from any cause. Patients lost to follow-up or were alive without any progression at the time of the last assessment were censored. ORR was assessed by using computed tomography (CT) scan according to RECIST v1.1 [13]. 

Continuous variables were described by median and interquartile range (IQR) and categorical variables by number (percentage), overall, and according to treatment groups. The proportion of missing values was specified. 

To compare OS and PFS across the three treatment groups, among the baseline characteristics, we selected the following potential confounding factors: age, sex, tumor localization, primary tumor resected, previous treatment (either radiotherapy or chemotherapy), metastases delay (synchronous or metachronous), number of metastatic sites, and chemotherapy regimen (oxaliplatin-based or irinotecan-based). Multiple imputation by using chained equations with 10 imputed data sets was performed to handle missing data that were assumed to be missing at random for all covariates [14]. The outcome times and events were included in the imputation dataset [14,15]. Rubin’s rules were applied in all subsequent analyses to summarize effect estimates and variances from the 10 different analyses across multiple imputed datasets [15]. 

Subsequently, to limit selection bias, differences in potential confounding factors between the three groups were controlled with inverse probability of treatment weighting method (IPTW) [16,17]. The propensity score was defined as each patient’s probability of being treated and constructed on baseline, which were included in a logistic regression. Stabilized weights were calculated with p/π, in which π is the propensity score and p the probability of being treated without considering covariates. The level of balance between each pair of the three treatment groups in the weighted study population (pseudo-cohort obtained by weighting) and in the unweighted study population (original cohort) for all variables included in the IPTW analysis was verified by computing pairwise standardized differences [18]. The standardized difference compares the difference in means in units of the pooled standard deviation. A standard difference that is less than 0.1 was considered to indicate a negligible difference between treatment groups [18]. 

To compare OS and PFS across the three treatment groups in the weighted study population with IPTW, we performed Kaplan–Meier, logrank tests, and Cox models with only one explanatory variable, namely the treatment group. We computed hazard ratios and their 95% CI by using robust variance. The proportional hazards assumption was checked by using graphical diagnoses, based on scaled Schoenfeld residuals. In addition, to compare ORR across the three treatment groups, we performed logistic regression models with IPTW. Finally, exploratory analyses examined the heterogeneity of treatment effect according to tumor localization. All tests were two-tailed, and a P value of less than 0.05 was considered statistically significant. Analyses were conducted with R^®^ software version 3.6.3 [19]. 

## 3. Results

### 3.1. Patients

Eight hundred and thirty-five patients were analyzed: 401 patients in the immediate anti-EGFR group, 305 patients in the delayed anti-EGFR group, and 129 patients in the immediate anti-VEGF group. In the delayed anti-EGFR group, 164 patients had the anti-EGFR introduction at cycle 2, 61 at cycle 3, and 40 at cycle 4.

The baseline characteristics in the unweighted study population are described in Table 1. The overall population had a median age of 64 years old, with a majority of men (65.9%). They mainly had left or rectal cancer (81.4%), with a previous resection of the primary tumor (70.2%). Metastases were mostly synchronous (80.3%), with one site (48.4%) or more (51.6%). The patients mostly had oxaliplatin-based chemotherapy (69.3%) The three groups presented differences (i.e., standardized difference >0.1) concerning all characteristics described in Table 1, except for the primary tumor localization.

**Figure 1:**
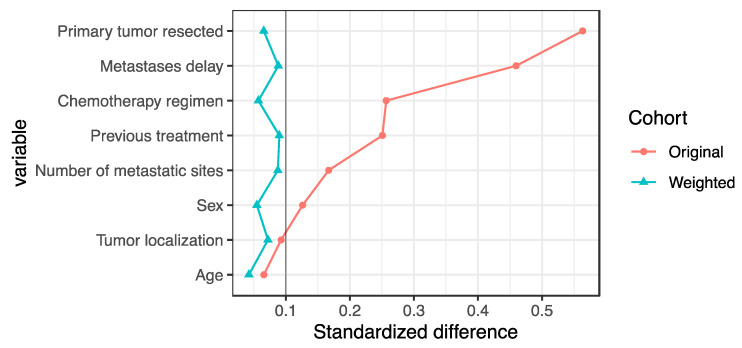
Standardized difference before and after propensity score weighting. The level of balance between the treatment groups was verified by computing the standardized differences. The standardized difference compares the difference in means in units of the pooled standard deviation. Three pairwise standardized differences were computed between the three treatment groups. A standard difference that is less than 0.1 has been taken to indicate a negligible difference between treatment groups.

After propensity scoring with IPTW was applied, there was no difference between the baseline characteristics of the three treatment groups; i.e., the standardized difference was less than 0.1 for all variables (Figure 1 and Table 2). 

### 3.2. Overall Survival

The median follow-up was 24.3 months (interquartile range: 22.0–25.9) with 309 events. A Kaplan–Meier estimate of OS according to treatment groups in the unweighted population is available in Appendix A. There was no overall difference concerning OS between the three groups in the unweighted population (log-rank, *p* = 0.071). 

**Figure 2:**
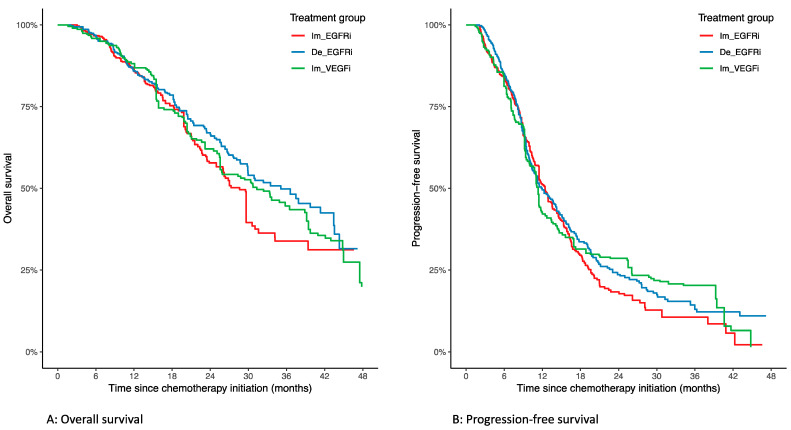
Survival according to treatment groups in the weighted study population. (**A**) Overall survival; (**B**) progression-free survival. Im_EGFRi = immediate anti-EGFR; De_EGFRi = delayed anti-EGFR; Im_VEGFi = immediate anti-VEGF.

In the weighted population, median OS was 28.6 months (95% CI: 23.5–34.1) in the immediate anti-EGFR group, 35.1 months (95% CI: 29.9–43.5) in the delayed anti-EGFR group, and 32.4 months (95% CI: 25.4–44.8) in the immediate anti-VEGF group. A Kaplan–Meier estimate of OS according to treatment groups in the weighted population is available in Figure 2A. There was no overall difference concerning OS between the three groups in the weighted population (weighted log-rank, *p* = 0.24). There was also no difference in OS when comparing groups in pairwise comparison (Table 3).

### 3.3. Progression-Free Survival

Median follow-up was 25.5 months (interquartile range: 22.3–29.0) with 567 events. A Kaplan–Meier of PFS according to treatment groups in the unweighted population is available in Appendix A. There was no overall difference concerning PFS between the three groups in the unweighted population (log-rank, *p* = 0.301). 

In the weighted population, median PFS was 12.4 months (95% CI: 11.5–14.5) in the immediate anti-EGFR group, 12.0 months (95% CI: 10.5–14.3) in the delayed anti-EGFR group, and 11.3 months (95% CI: 9.4–14.6) in the immediate anti-VEGF group. A Kaplan–Meier of PFS according to treatment groups in the weighted population is available in Figure 2B. There was no overall difference concerning PFS between the three groups in the weighted population (weighted log-rank, *p* = 0.56). There was also no difference in PFS when comparing the groups in pairwise comparison (Table 3).

### 3.4. Objective Response Rate

In the weighted population, the ORR was 45.2% (N = 154/341) in the immediate anti-EGFR group, 58.6% (N = 168/286) in the delayed anti-EGFR group, and 50.1% (N = 55/109) in the immediate anti-VEGF group. The ORR was similar between the immediate anti-VEGF group and the immediate anti-EGFR group (aOR 1.24; 95% CI: 0.71–2.19) and between the immediate anti-VEGF group and the delayed anti-EGFR group (aOR 0.71; 95% CI: 0.41–1.25). However, the ORR was higher in the delayed anti-EGFR group than in the immediate anti-EGFR group (aOR 1.75; 95% CI: 1.20–2.54) (Table 3).

### 3.5. Tumor Localization

No significant difference between anti-EGFR and anti-VEGF treatment concerning OS, PFS, and ORR was identified in the subgroups of right (right + transverse tumor) and left (left + rectal tumor) tumors in the weighted study population (Table 3).

## 4. Discussion

In this study, the strategy of delaying the introduction of an anti-EGFR to cycle 2 to 4 of doublet chemotherapy was non deleterious on OS compared to the immediate introduction of an anti-VEGF or anti-EGFR. The median OS was 35.1 months in the delayed anti-EGFR group, compared to 28.6 months in the immediate anti-EGFR group, and 32.4 months in the immediate anti-VEGF group, with nonsignificant differences. In addition, there was no significant difference concerning PFS and ORR between the three groups.

Delaying the introduction of an anti-EGFR is common in real-life clinical practice. Thus, in this study, 56.8% patients had an immediate introduction of an anti-EGFR at cycle 1, 23.2% had a delayed introduction at cycle 2, and 20.0% had a delayed introduction at cycle 3 or 4. No study had ever compared this strategy to both the immediate introduction of anti-EGFR or anti-VEGF. This large and propensity-weighted study suggests that this strategy of delaying anti-EGFR introduction while waiting for *RAS* status is non-deleterious.

The median OS of the immediate introduction groups is in line with the literature. The median OS in the immediate anti-VEGF group of 32.4 months is very close to the median OS of 31.2 months of CALGB/SWOG 80405 phase III trial [3] and slightly higher than in the FIRE-3 phase III trial [20] and PEAK phase II trial [21] (respectively, 25.0 and 28.8 months). The median OS in the immediate anti-EGFR group of 28.6 months is close to the median OS found in the three previous trials, with a median OS of 32.0, 33.1, and 41.3 months respectively. 

A median PFS of 12.4 months in the immediate anti-EGFR group is also concordant with the literature (10.3 months in FIRE-3, 11.4 in CALGB/SWOG 80405, and 13.0 in PEAK), as is the median PFS of 11.3 months in the immediate anti-VEGF group (10.2 months in FIRE-3, 11.3 in CALGB/SWOG 80405, and 9.5 in PEAK). The trend of longer survival in delayed versus immediate anti-EGFR group found for OS was not found for PFS.

The ORR was slightly inferior to that in the existing literature. The ORR was 45.2% in the immediate anti-EGFR group (versus 65% in FIRE-3, 57.8% in PEAK, and 59.6% in CALGB/SWOG 80405) and 50.1% in the immediate anti-VEGF group (versus 60% in FIRE-3, 53.5% in PEAK, and 55.2% in CALGB/SWOG 80405). This could be explained by the fact that the timing of the CT scan was not available in the registry-based study. It could have resulted in a heterogeneity in the evaluation with later evaluations in routine clinical practice rather than in trials, which results in underestimating ORR [22]. Surprisingly, the ORR in the delayed anti-EGFR group is higher than in the immediate anti-EGFR group. A potential explanation is that the treatment-based Czech registry did not include patients with early disease progression during first-line chemotherapy or patients who did not tolerate chemotherapy before the addition of anti-EGFR. This could have led to a higher ORR in the delayed anti-EGFR group. 

No difference was identified according to the side (right versus left) of mCRC for the response to the anti-EGFR versus anti-VEGF. It had previously been shown by a large meta-analysis that patients with *RAS* wild-type left CRC had a greater survival benefit from anti-EGFR treatment compared with anti-VEGF treatment when added to standard chemotherapy [23]. This is also discordant with the results of the original French study in which a benefit on PFS of the anti-EGFR was found for left-sided mCRC. This may be due to the median PFS for delayed anti-EGFR in the French study being higher than in this study: 13.8 versus 12 months, respectively.

The main limitation of this study is its retrospective nature. The registry-based origin of the Czech cohort led to the absence of some covariates, such as *BRAF*, microsatellite instability status, ECOG performance status, dose intensity, subsequent therapies, side effects, and timing of CT scans. The *BRAF* WT status was available for only about 40% of patients in the Czech cohort and in all of the patients of the French cohort. Thus, it could not be included in the propensity score and could have led to a bias due to its prognostic impact. The absence of randomization leading to the initial heterogeneity between groups has been well balanced by propensity score weighting, but some prognostic factors could, therefore, not be included in the propensity score. The different origins and timing of enrollment of the two initial cohorts remain a bias of this study. Moreover, the exclusion of patients in the Czech cohort with early disease progression during the first-line chemotherapy and of those who did not tolerate chemotherapy before the addition of an anti-EGFR could have led to a nonsignificant better outcome in OS in the delayed anti-EGFR group. Missing data due to the retrospective nature were handled by multiple imputation by using chained equations.

This study underlines the difficulty of quickly obtaining *RAS* status results [10]. The need for outsourcing the samples to an external molecular biology platform and the time required for *RAS* status determination are potential explanations. Circulating tumor DNA should help to address this issue. Mean and median turnaround times of tissue-based methods are 13 days and 11 days, respectively, versus 2 days for plasma-based methods. The concordance rate of *RAS* status in plasma and tumor tissue varies according to studies and techniques, but can be up to 95% [24,25,26].

## 5. Conclusions

In conclusion, this large propensity-weighted study suggests that delaying anti-EGFR introduction while waiting for *RAS* status is non-deleterious on OS, PFS, and ORR, compared to its immediate introduction or to the immediate introduction of anti-VEGF.

## Figures and Tables

**Table 1:** Baseline characteristics in the unweighted study population.

Item	Overall	Immediate Anti-EGFR	Delayed Anti-EGFR	Immediate Anti-VEGF	Standardized Difference *	Missing (%)
Number of patients	835	401	305	129		
Age, median (range), years	64.0 [56.6, 69.7]	64.0 [56.6, 68.9]	63.9 [56.5, 70.1]	64.0 [57.3, 73.8]	0.145	0.0
Sex					0.126	0.0
Men	550 (65.9)	278 (69.3)	184 (60.3)	88 (68.2)		
Women	285 (34.1)	123 (30.7)	121 (39.7)	41 (31.8)		
Tumor localization					0.093	3.4
Right/transverse	150 (18.6)	66 (17.1)	64 (21.5)	20 (16.1)		
Left/Rectum	657 (81.4)	320 (82.9)	233 (78.5)	104 (83.9)		
Primary tumor resected					0.563	0.0
No	249 (29.8)	66 (16.5)	113 (37.0)	70 (54.3)		
Yes	586 (70.2)	335 (83.5)	192 (63.0)	59 (45.7)		
Previous treatment					0.251	0.0
No	557 (66.7)	235 (58.6)	224 (73.4)	98 (76.0)		
Yes	278 (33.3)	166 (41.4)	81 (26.6)	31 (24.0)		
Metastases delay					0.460	26.0
Synchronous	496 (80.3)	222 (91.0)	192 (78.4)	82 (63.6)		
Metachronous	122 (19.7)	22 (9.0)	53 (21.6)	47 (36.4)		
Metastatic sites					0.167	30.8
1	280 (48.4)	100 (45.0)	125 (55.1)	55 (42.6)		
>=2	298 (51.6)	122 (55.0)	102 (44.9)	74 (57.4)		
Chemotherapy regimen					0.257	0.1
Oxaliplatin-based	578 (69.3)	263 (65.6)	237 (78.0)	78 (60.5)		
Irinotecan-based	256 (30.7)	138 (34.4)	67 (22.0)	51 (39.5)		
Country					NA	0.0
Czech Republic	573 (68.6)	401 (100.0)	172 (56.4)	0 (0.0)		
France	262 (31.4)	0 (0.0)	133 (43.6)	129 (100.0)		

* The level of balance between the treatment groups was verified by computing the standardized differences. The standardized difference compares the difference in means in units of the pooled standard deviation. Three pairwise standardized differences were computed between the three treatment groups. A standard difference that is less than 0.1 has been taken to indicate a negligible difference between treatment groups.

**Table 2:** Baseline characteristics in the weighted study population.

Item	Overall	Immediate Anti-EGFR	Delayed Anti-EGFR	Immediate Anti-VEGF	Standardized Difference *	Missing (%)
Number of patients	833	409	310	114		
Age, years					0.04	0.0
<60 years	445 (53.5)	214 (52.3)	168 (54.3)	63 (55.4)		
≥60years	388 (46.6)	195 (47.7)	142 (45.7)	51 (44.6)		
Sex					0.05	0.0
Men	542 (65.1)	267 (65.3)	205 (66.0)	71 (62.1)		
Women	291 (34.9)	142 (34.7)	105 (34.0)	43 (38.0)		
Tumor localization					0.07	0.0
Right/transverse	154 (18.5)	72 (17.7)	57 (18.2)	25 (22.1)		
Left/Rectum	679 (81.5)	336 (82.3)	253 (81.8)	89 (78.0)		
Primary tumor resected					0.07	0.0
No	259 (31.1)	129 (31.5)	91 (29.4)	39 (34.0)		
Yes	574 (68.9)	280 (68.5)	219 (70.6)	75 (66.0)		
Previous treatment					0.09	0.0
No	556 (66.8)	271 (66.3)	203 (65.5)	82 (71.8)		
Yes	277 (33.2)	138 (33.7)	107 (34.5)	32 (28.2)		
Metastases delay					0.09	0.0
Synchronous	617 (74.1)	302 (73.9)	235 (75.9)	80 (70.0)		
Metachronous	216 (25.9)	107 (26.1)	75 (24.1)	34 (30.0)		
Metastatic sites					0.09	0.0
1	405 (48.6)	201 (49.2)	154 (49.7)	49 (43.2)		
≥2	428 (51.4)	208 (50.8)	156 (50.3)	65 (56.8)		
Chemotherapy regimen					0.06	0.0
Oxaliplatin-based	594 (71.3)	292 (71.3)	218 (70.3)	85 (74.1)		
Irinotecan-based	239 (28.7)	117 (28.7)	92 (29.7)	30 (25.9)		

* The level of balance between the treatment groups was verified by computing the standardized differences. The standardized difference compares the difference in means in units of the pooled standard deviation. Three pairwise standardized differences were computed between the three treatment groups. A standard difference that is less than 0.1 has been taken to indicate a negligible difference between treatment groups.

**Table 3:** Regression models in the weighted study population.

	Cox Models					
	**Delayed Versus Immediate Anti-EGFR**		**Immediate Anti-VEGF** **Versus Immediate Anti-EGFR**		**Immediate Anti-VEGF** **Versus Delayed Anti-EGFR**	
	aHR (95%CI)	*p*	aHR (95%CI)	*p*	aHR (95%CI)	*p*
**Overall survival**	0.78 [0.60–1.01]	0.06	0.92 [0.69–1.24]	0.61	1.19 [0.88–1.60]	0.26
Right/transverse tumor	1.03 [0.59–1.79]	0.93	0.86 [0.45–1.64]	0.64	0.83 [0.43–1.60]	0.58
Left/Rectum tumor	0.71 [0.53–0.97]	0.03	0.89 [0.63–1.25]	0.49	1.24 [0.87–1.75]	0.23
**Progression-free survival**	0.91 [0.75–1.10]	0.32	0.93 [0.73–1.18]	0.55	1.02 [0.80–1.31]	0.83
Right/transverse tumor	0.89 [0.57–1.40]	0.62	0.84 [0.48–1.46]	0.53	0.94 [0.53–1.64]	0.82
Left/Rectum tumor	0.91 [0.74–1.13]	0.40	0.92 [0.70–1.21]	0.57	1.01 [0.77–1.33]	0.92
	Logistic Regression Models					
	**Delayed Versus Immediate Anti-EGFR**		**Immediate Anti-Vegf Versus Immediate Anti-EGFR**		**Immediate Anti-VEGF Versus Delayed Anti-EGFR**	
	aOR (95%CI)	*p*	aOR (95%CI)	*p*	aOR (95%CI)	*p*
**Objective response rate**	1.75 [1.20–2.54]	0.004	1.24 [0.71–2.19]	0.45	0.71 [0.41–1.25]	0.23
Right/transverse tumor	1.69 [0.67–4.28]	0.26	0.92 [0.21–3.98]	0.91	0.54 [0.12–2.42]	0.42
Left/Rectum tumor	1.78 [1.17–2.68]	0.007	1.38 [0.77–2.46]	0.28	0.78 [0.43–1.40]	0.40

aHR/aOR (95%CI): hazard ratio/odds ratio (95% confidence interval) adjusted by inverse probability of treatment weighting for the following: age, sex, tumor localization, primary tumor resected, previous treatment (either radiotherapy or chemotherapy), metastases delay (synchronous or metachronous), number of metastatic sites, and chemotherapy regimen (oxaliplatin-based or irinotecan-based).

**Figure A1:**
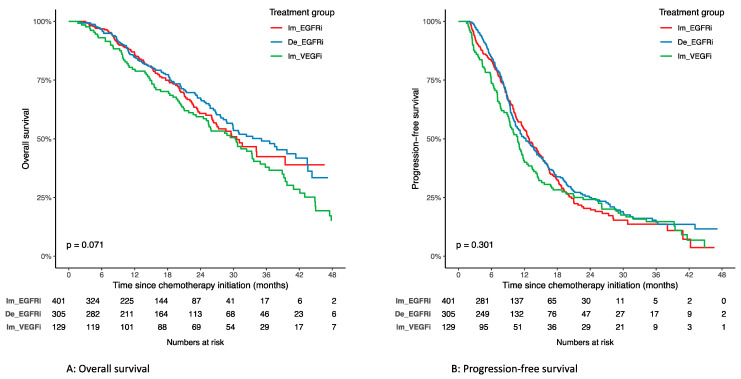
Survival according to treatment groups in the unweighted study population. (**A**) Overall survival; (**B**) progression-free survival. Im_EGFRi = immediate anti-EGFR; De_EGFRi = delayed anti-EGFR; Im_VEGFi = immediate anti-VEGF.

## Data Availability

The data presented in this study are available upon request from the corresponding author.
